# Pharmacodynamic analysis of tumour perfusion assessed by ^15^O-water-PET imaging during treatment with sunitinib malate in patients with advanced malignancies

**DOI:** 10.1186/2191-219X-2-31

**Published:** 2012-06-09

**Authors:** Andrew M Scott, Paul L Mitchell, Graeme O'Keefe, Timothy Saunder, Rodney J Hicks, Aurora Poon, Charles Baum, Nicoletta Brega, Timothy J McCarthy, Guy C Toner

**Affiliations:** 1 Centre for PET, Ludwig Institute for Cancer Research and Ludwig Oncology Unit, Austin Hospital, Studley Road, Heidelberg, Victoria, 3084, Australia; 2 Division of Cancer Medicine and Centre for Molecular Imaging, Peter MacCallum Cancer Centre, Melbourne, Victoria, 8006, Australia; 3 Pfizer Oncology, La Jolla, San Diego, CA, 92037, USA; 4 Pfizer Oncology, Milan, 20100, Italy; 5 Pfizer Oncology, Groton, CT, 06340, USA

**Keywords:** sunitinib, tumour perfusion, FDG-PET.

## Abstract

**Background:**

We evaluated pharmacodynamic changes in tumour perfusion using positron emission tomography (PET) imaging with ^15^O-water to assess biological response to sunitinib, a multitargeted tyrosine kinase inhibitor.

**Methods:**

Patients with advanced malignancies received sunitinib 50 mg/day orally, once daily for 4 weeks on treatment, followed by 2 weeks off treatment, in repeated 6-week cycles. Quantitative measurement of tumour perfusion was assessed using ^15^O-water-PET at baseline and after 2 weeks of treatment. At least one reference tumour lesion was included in the fields of view and assessed at both time points. Patients also underwent ^18^ F-fluorodeoxyglucose (FDG)-PET imaging at baseline and after 2 and 4 weeks of treatment. Radiological response of the reference tumour lesion and overall radiological response were assessed at week 12. Serum pharmacokinetic and biomarker analyses were also performed.

**Results:**

Data were available for seven patients. Compared with baseline, all patients experienced a decrease in reference tumour blood flow ranging from 20 % to 85 % and also a reduction in the FDG standard uptake value ranging from 29 % to 67 %. Six patients experienced a partial metabolic response based on FDG-PET criteria. Four patients had stable disease defined by radiological response (Response Evaluation Criteria in Solid Tumors) lasting between 4 and 12 cycles. An association between perfusion change and clinical benefit, and biomarker levels including vascular endothelial growth factor was observed.

**Conclusion:**

Administering sunitinib to patients with advanced malignancies is associated with early biological responses, including decreased blood flow in secondary tumour deposits.

## Background

Once solid tumours have reached an advanced or metastatic stage, treatment is largely palliative rather than curative, with the aim of prolonging survival, relieving pain and other symptoms, and maintaining or improving quality of life [1-3]. Pharmacotherapy at such advanced stages is generally targeted at slowing, halting, or - if possible - reversing tumour progression, and at reducing the chance of further metastasis.

Targeted biological therapies with antiangiogenic properties may be particularly beneficial in the palliative care setting by preventing the neovascularisation required for tumour progression beyond a certain size [4]. The early and more subtle biological effects of new antiangiogenic agents may not be detected by conventional measures of tumour response, which are based on changes in tumour size. Positron emission tomography (PET) imaging assesses functional aspects of tumour status and may detect biological responses (e.g. decreased metabolic rate and blood flow) before changes in tumour size are apparent with conventional imaging techniques [5,6].

Sunitinib (Sutent®; Pfizer Inc., New York, NY, USA) is an orally active, multitargeted tyrosine kinase inhibitor with both antitumour and antiangiogenic effects. Its targets include vascular endothelial growth factor receptors 1, 2, and 3 (VEGFRs-1, -2, and −3), platelet-derived growth factor receptors α and β, stem-cell factor receptor, FMS-like tyrosine kinase 3, and glial cell line-derived neurotrophic factor receptor [7-12]. Sunitinib is approved in many countries for the treatment of gastrointestinal stromal tumours after disease progression on or intolerance to imatinib mesylate therapy [13], for the treatment of advanced renal cell carcinoma [14], and for the treatment of advanced pancreatic neuroendocrine tumours [15].

In a pilot study of 55 adults with advanced malignancies treated with sunitinib, biological activity and antitumour effects were seen in a broad range of tumour types [16]. Seven patients continued therapy for more than 12 months, and two patients remained on therapy more than 3 years after enrolment. Two types of antitumour activity were seen: tumour shrinkage and central tumour necrosis. Serial PET imaging detected ≥20 % reductions in the standard uptake value (SUV) of ^18^ F-fluoro-2-deoxy-D-glucose (FDG) as early as the second week of treatment. Both PET response and clinical benefit correlated with trough plasma levels of sunitinib and its principal metabolite (SU12662). The present paper reports on a subset of seven patients from this study who, in addition to standard study procedures, also underwent pharmacodynamic assessment with quantitative ^15^O-water-PET imaging to assess the effects of sunitinib treatment on tumour perfusion.

## Methods

### Patients and treatment

The use of FDG-PET and other PET-based imaging techniques to assess biological response to sunitinib was investigated in a 12-week, open-label, prospective pilot study involving four groups. The present study describes the group of patients who underwent ^15^O-water- and FDG-PET imaging. All patients were ≥18 years old, with radiological evidence of metastatic or advanced malignancy for which there was no available therapy with curative potential. Other key eligibility criteria included Karnofsky performance status > 60 %, life expectancy > 12 weeks without rapidly progressive disease, and adequate liver function (aspartate transaminase (AST) or alanine transaminase (ALT) ≤ 2.5 × upper limit of normal (ULN); AST or ALT ≤ 5.0 × ULN or bilirubin ≤ 1.5 × ULN if liver function abnormalities were due to underlying malignancy), renal function (serum creatinine ≤ 1.5 × ULN or calculated creatinine clearance > 40 mL/min by the Cockcroft-Gault formula), and bone marrow function (absolute neutrophil count (ANC) ≥ 1.5 × 10^9^/L, platelets ≥ 75 × 10^9^/L, and haemoglobin > 10 g/L; ANC ≥ 1.0 × 10^9^/L, platelets ≥ 50 × 10^9^/L, or haemoglobin > 10 g/L for patients with haematological malignancies and bone marrow involvement). At screening, all patients had to have at least one tumour mass detectable by FDG-PET imaging that was suitable for serial quantitative assessment and had not been included in a prior radiotherapy field. Female patients were postmenopausal, surgically sterile, or using effective contraception. Key exclusion criteria included the use of any other anticancer agent or investigational agent within 4 weeks prior to the start of sunitinib treatment, prior specific anti-vascular endothelial growth factor (VEGF) treatment, and any condition requiring treatment that was likely to affect the reliability of the serial PET assessments. Patients were also excluded if they had a medical or psychiatric condition that would limit full compliance with the study. Concomitant treatments necessary for the patients' well-being were given at the discretion of the investigator. The study was carried out in accordance with the International Conference on Harmonisation Good Clinical Practice guidelines and was approved by an institutional review board. All patients provided written informed consent.

Patients received oral sunitinib 50 mg once daily for 4 weeks on treatment, followed by 2 weeks off treatment (schedule 4/2) in repeated 6-week cycles. The primary study period was the first two cycles (12 weeks) of treatment. Patients benefiting from the treatment were permitted to receive additional cycles of sunitinib for up to 1 year. The study drug was discontinued in patients exhibiting grade 4 haematological toxicity, grade 3 thrombocytopenia with haemorrhage of grade 3/4, or grade 3/4 non-haematological toxicity, and only restarted when the ANC recovered to ≥ 1.5 × 10^9^/L and platelet count to 75 × 10^9^/L.

### Study assessments

#### *Tumour perfusion*

Tumour perfusion was assessed using a radioactive tracer (^15^O-water) and PET imaging. This technique allowed quantification of the arterial concentration of ^15^O-water and calculation of the perfusion rate. Quantification of blood flow within the tumour (expressed in milliliter per gram per minute) was performed.

^15^O-water-PET imaging was performed at baseline and after 2 weeks of treatment using an ECAT 951/31R scanner ( CTI PET Systems, Knoxville, TN, USA), employing 16 rings of bismuth germanate oxide block detectors with an axial extent of 108 mm and a spatial resolution of 5.4 mm at the scanner's centre of field of view. At least one viable reference tumour lesion identified by FDG-PET scanning was included in the field of view and was assessed at both time points. Emission scans were acquired dynamically in three-dimensional (3D) mode during bolus intravenous injections of 370 MBq of ^15^O-water into the antecubital vein. The dynamic acquisition consisted of 11 frames acquired over 3 min with 4 × 5-s, 2 × 10-s, and 4 × 30-s frames. A transmission image (10-min acquisition) was also obtained. Dynamic dataset images were reconstructed with both 3D-filtered back-projection, using a 3D reprojection algorithm (3D-FORE/FBP), and 3D attenuation-weighted ordered-subsets expectation maximisation (AW-OSEM3D) [17,18]. As a result of the superior image quality of the AW-OSEM3D reconstruction, this was used for the subsequent analysis.

#### *Glucose uptake*

All patients had an FDG-PET scan at screening, up to 14 days prior to commencing sunitinib treatment. Further FDG-PET scans were performed at baseline and after 2 and 4 weeks of treatment, with SUV measurement of the glucose metabolism of both reference and overall tumour lesions. Whole body FDG-PET scanning was performed using a Philips Allegro dedicated PET scanner (Philips Medical Systems, Cleveland, Ohio, USA) under standardised conditions, following an overnight fast, and with imaging commencing exactly 60 min after FDG administration. All studies were performed with measured attenuation correction and were reconstructed using RAMLA 3D [19] and reconstructed in units of SUV. Blood glucose and lean body mass were recorded. Overall response (OR) was assessed in patients completing at least 4 weeks of treatment with all assessments performed. OR was defined as a ≥20 % reduction in FDG uptake as assessed by SUV corrected for lean body mass in all lesions present at baseline in the absence of any new lesions. Progressive disease (PD) was defined as a ≥15 % increase in FDG uptake in any lesion or emergence of any new lesions. A mixed response was defined as multiple lesions with an OR or PD, plus other lesions with stable disease.

#### *Image analysis of tumour perfusion*

**Figure 1:**
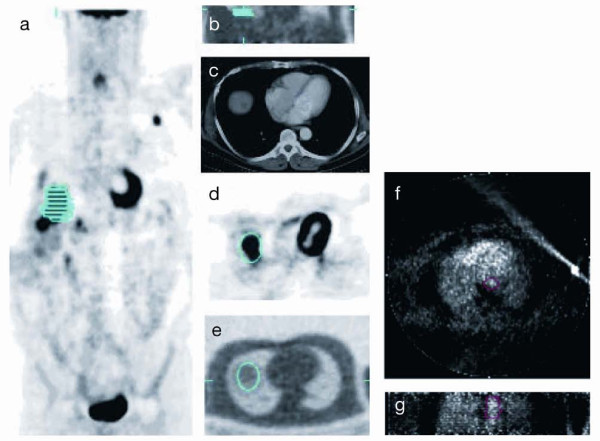
**Reference tumour volume of interest using the attenuation image for the FDG and **^15^**O-water studies.** Liver metastasis defined on the FDG-PET emission image ((**a**) coronal and (**d**) transaxial), also seen on the CT scan (**c**), was identified and transposed onto the ^15^O-water acquired attenuation image ((**b**) coronal and (**e**) transaxial) to be subsequently applied to the dynamic ^15^O-water emission image. The descending aorta volume of interest markup from ^15^O-water early dynamic frames ((**f**) transaxial and (**g**) coronal).

The volume of interest (VOI) for the reference tumour for each patient was defined using the FDG-PET data by thresholding to approximately 75 % of the maximum SUV in tumour and also by comparing to tumour size on computed tomography scan. The VOIs were defined for the baseline and 2-week post-treatment ^15^O-water studies using the associated baseline and 2-week post-treatment FDG scan-determined VOIs. The entire VOI was used to determine the activity for ^15^O-water. By using the attenuation image for the FDG and ^15^O-water studies, the emission images from both studies were co-registered so that the reference tumour VOIs could be applied to the water study (Figure 1). A summed dynamic image from the ^15^O-water study was then used to define the descending aorta. The resultant tumour and aortic VOIs were used to determine the integrated activity for their respective regions, which then defined the response and input functions, respectively.

#### *Spectral analysis*

**Figure 2:**
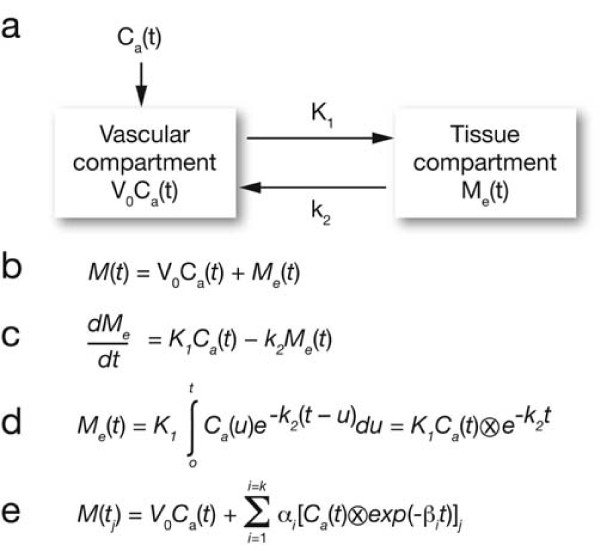
**Spectral analysis model and operational equations.** Spectral analysis model used to calculate tumour blood flow comprising vascular and tissue compartments (**a**), and the operational equation defining tissue blood flow (**b**) and (**c**) where *M*(*t*) denotes the detected activity within a volume of interest and is made up of a vascular component given by *V*_0_*C*_a_(*t*) and an extra-vascular or tissue component *M*_e_(*t*), where *V*_0_ denotes the vascular volume term, and *C*_a_(*t*), the arterial input term. Such a system is governed by a so-called one-tissue-compartment model dictated by the equation given in (c). The analytic solution to this differential equation is expressed in (**d**). The equation in (**e**) illustrates the linearisation process whereby a spectrum of exponentials convolved with the input function are predetermined so as to allow the linear solution for the blood-perfusion weighting terms given by *α*_i_.

A one-tissue compartment model was used to calculate the tumour blood flow and comprised vascular and tissue compartments (Figure 2a). The operational equation defining tissue blood flow is given in Figure 2b,c, where *M*(*t*) denotes the detected activity within a VOI and is made up of a vascular component given by *V*_0_*C*_a_(*t*) and an extra-vascular or tissue component *M*_e_(*t*), where *V*_0_ denotes the vascular volume term, and *C*_a_(*t*), the arterial input term. Such a system is governed by a so-called one-tissue-compartment model dictated by the equation given in Figure 2c, where *K*_1_ denotes the blood perfusion from vascular to tissue compartment, and *k*_2_, the washout rate, as illustrated in Figure 2. The analytic solution to this differential equation is expressed in Figure 2d. Non-linear analytical techniques are adopted to solve for the rate constants *K*_1_ and *k*_2_.

For the purposes of this analysis, tumour blood perfusion was derived from this equation by using the tumour VOI to input data into the tissue compartment of this model. Normally, the vascular compartment is neglected in this model as it generally makes a small contribution (approximately 5 %) to the net activity, thus reducing the analysis to a single-compartment model. However, for tumour regions, the high vascular density requires this term to be explicitly modelled [20]. Thus, both tissue perfusion and blood volume were determined. Spectral techniques were adopted in the analysis of tumour blood flow data due to the robustness of the linear approach and the input/response function delays. The equation in Figure 2e illustrates the linearisation process whereby a spectrum of exponentials convolved with the input function are predetermined so as to allow the linear solution for the blood-perfusion weighting terms given by *α*_i_. By adopting such a spectrum of exponentials, the problem is linearised and, so, is less prone to noise effects in the measured data. The vascular term is incorporated implicitly by ranging the spectrum of exponentials from *β*_i_ = 0 to a predetermined maximum flow rate. Upon solving this system using standard linear techniques, the blood perfusion + vascular volume of the tumour is determined by summing the spectrum of *α*_i_ components.

#### *Response assessment*

Radiological response of the reference tumour lesion and overall radiological response were assessed at week 12 using Response Evaluation Criteria in Solid Tumors (RECIST) [21]. Clinical benefit was deemed to be present if the treating clinician judged that the patient was benefiting from treatment (RECIST-defined stable disease or response) and continued the therapy beyond the 12-week study period. Formal response evaluation and clinical benefit assessment were performed every 12 weeks for patients continuing to receive sunitinib after the second cycle of treatment.

#### *Pharmacokinetic and biomarker analyses*

Blood samples for analysis of pharmacokinetic parameters were collected on day 1 and every 14 days in cycle 1. Levels of sunitinib and its principal metabolite, SU12662, were determined using liquid chromatography and mass spectrometry techniques. Peripheral blood for biomarker analysis was collected before and 6 h after the first dose on day 1 and at every visit thereafter (i.e., every 14 days for the first 12 weeks). Samples were screened by enzyme-linked immunosorbent assays and 2D-gel electrophoresis for proteins whose levels may be altered in association with sunitinib activity or exposure: for example, VEGF and soluble VEGFR-2 (sVEGFR-2).

#### *Statistical analysis*

Data were summarised using descriptive statistics. Analysis of change in tumour perfusion or FDG-SUV was performed using the Student's *t* test, comparing patients with clinical benefit to those without clinical benefit of treatment (one-sided *t* test, assumed unequal variance). Analysis of day 15 trough levels of sunitinib plus SU12662 was performed using a *z*-test for patients with clinical benefit and patients without clinical benefit and a reference of 50 ng/mL. Linear relationships between change in tumour perfusion or change in FDG-SUV and biomarker levels were determined using Pearson's product moment correlation with 95 % confidence intervals.

## Results

**Figure 3:**
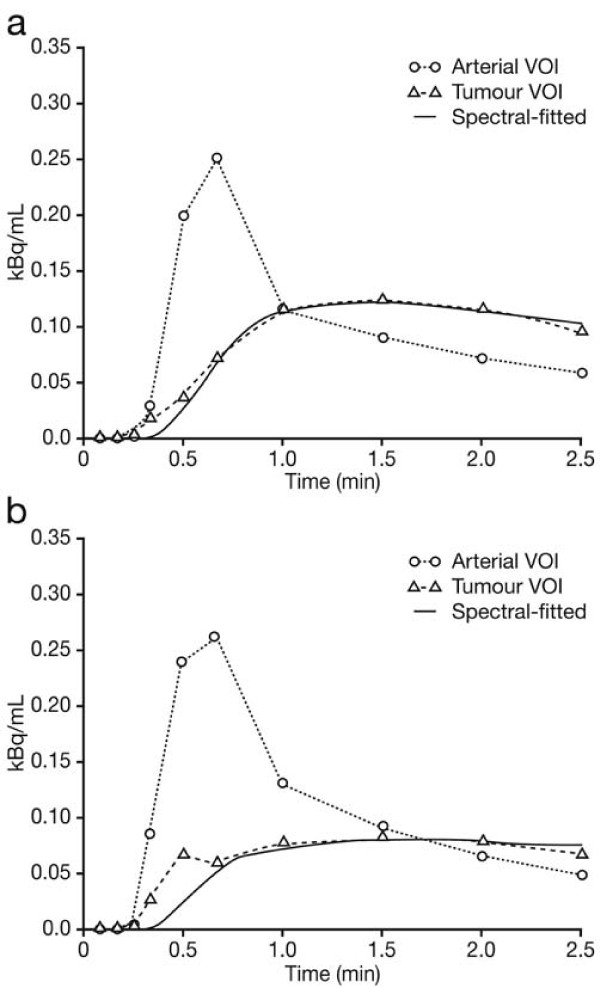
**Tumour blood flow analysis in patient 4.** The result of the spectral fitting procedure for patient 4 pre-treatment (**a**) and post-treatment (**b**), with the associated estimation of tumour blood perfusion reported in the value of *K*_1._

Data are reported for seven patients who underwent all study investigations and completed at least two treatment cycles (Table 1). Dosing was delayed for patients 2 and 3 before starting cycle 2 of therapy, while the cycle 2 dose of patient 7 was reduced by 25 %. Figure 3 illustrates the result of the spectral fitting procedure for patient 4 for pre- and post-treatment, with the associated estimation of tumour blood reported in the value of *K*_1_. Partial volume effects were not considered as the tumour VOIs were of similar size for both pre- and post-treatment.

**Table 1:** Response among patients with advanced malignancies treated with sunitinib 50 mg/day on schedule 4/2

**Patient**	**Primary tumour type**	**Reference tumour site**	**Change in perfusion at week 2 (%)**	**Change in FDG-SUV in reference tumour at week 2 (%)**	**Overall FDG-PET response at week 4**	**Radiological response in reference lesion at week 12**	**Overall radiological response at week 12**	**Duration of treatment (number of 6-week cycles)**	**Clinical benefit**
1	Renal	Right hepatic lobe	−85	−39	Yes	SD	SD	4	Yes
2	Colon	Right hepatic lesion	−77	−37	Yes	SD	SD	6	Yes
3	Colon	Right hepatic lobe	−59	−33	Yes	PD	PD	2	No
4	Colon	Right hepatic lobe	−34	−44	Yes	PD	PD	2	No
5	NSCLC	Left lung lesion	−69	−67	Yes	SD	SD	12	Yes
6	Colon	Right hepatic lesion	−38	−32	Yes	SD	SD	7	Yes
7	Oesophageal	Right hepatic dome	−20	−29	No	PD	PD	2	No

Biological (week 2) and radiological (week 12) responses among patients with advanced malignancies treated with sunitinib 50 mg given once daily for 4 weeks on treatment followed by 2 weeks off treatment (schedule 4/2). Clinical benefit was deemed to be present if the treating clinician judged that the patient was benefiting from treatment and continued therapy beyond the 12-week study period. D15:D1, day15:day1 ratio; FDG-PET, ^18^ F-fluorodeoxyglucose positron emission tomography; FDG-SUV, ^18^ F-fluorodeoxyglucose standard uptake value; NSCLC, non-small cell lung cancer; PD, progressive disease; SD, stable disease.

Table 1 details the site of the primary and reference tumours as well as the biological and radiological response of the reference tumour and overall tumour response to sunitinib therapy. All patients exhibited a decrease in reference tumour blood flow, ranging from 20 % to 85 %, and a reduction in FDG-SUV from baseline in the reference tumour, ranging from 29 % to 67 %. Six of the seven patients experienced a partial metabolic response at week 4 based on FDG-PET criteria. Four patients had stable disease lasting between four cycles (24 weeks) and 12 cycles (72 weeks) confirmed by RECIST at restaging.

**Figure 4:**
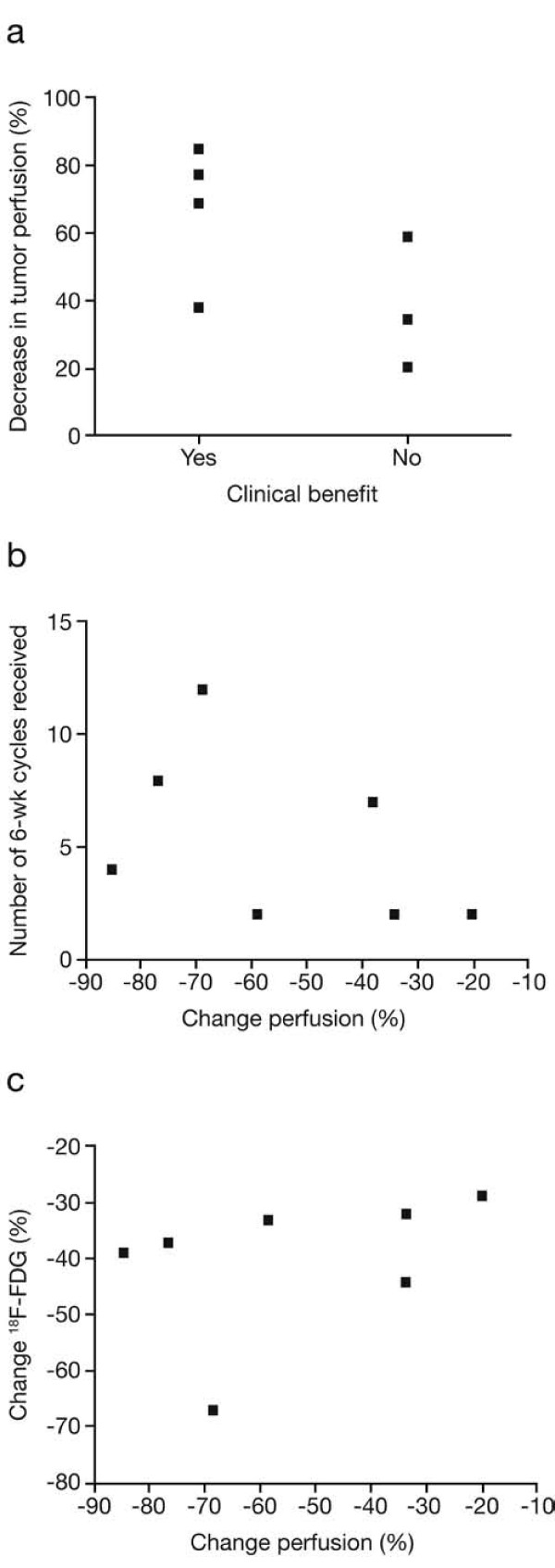
**Correlation between reduction in tumour perfusion and clinical benefit, duration of therapy, and FDG-SUV change.** Potential correlations between percent reduction in tumour perfusion and clinical benefit (*p* = 0.05) (**a**), duration of therapy (*p* = 0.05) (**b)**, and percent reduction in FDG-SUV (*p* > 0.05) (**c**).

**Figure 5:**
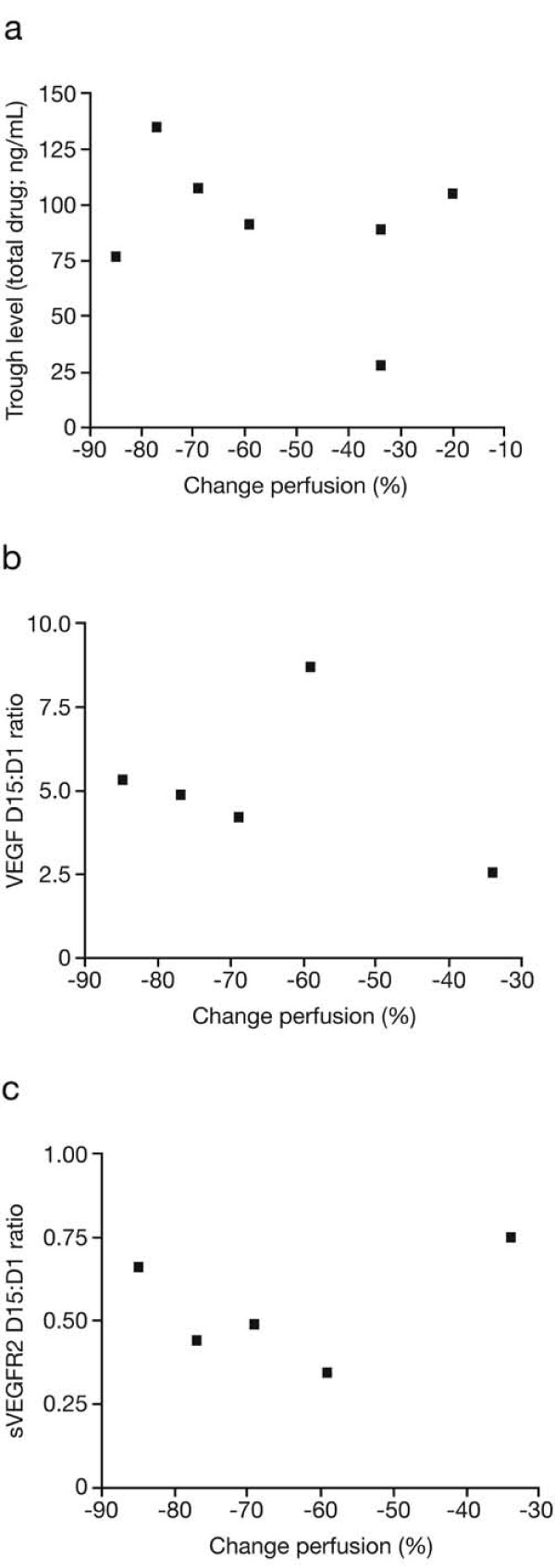
**Correlation between reduction in tumour perfusion and trough levels of total drug, VEGF, and sVEGFR-2.** Potential correlations between percent reduction in tumour perfusion and day 15 trough levels of total drug (sunitinib plus SU12662; *p* > 0.05) (**a**), change in plasma VEGF levels (*p* = 0.05) (**b**), and change in plasma sVEGFR-2 levels (*p* > 0.05) (**c**). D15:D1, day 15:day 1 ratio.

Further analyses were undertaken in order to investigate potential correlations between percent reduction in tumour perfusion and clinical benefit, duration of therapy, percent reduction in FDG-SUV, plasma levels of sunitinib and SU12662, and changes in plasma levels of VEGF and sVEGFR-2 (Table 2, Figures 4 and 5). There was a possible association between the degree of change in tumour perfusion measured after 2 weeks of therapy and subsequent clinical benefit (*t* test, *p* = 0.05; Figure 4a) as well as with duration of therapy (Figure 4b) and with change in biomarker levels, particularly VEGF (Figures 5b,c). A further non-significant trend was observed between VEGF biomarker levels and FDG-SUV (percent) change (sVEGFR2: *r* = 0.76, *p* > 0.05 and VEGF: *r* = −0.75, *p >* 0.05). In this study, there was no evidence of correlation between the degree of change in tumour perfusion with the degree of FDG-SUV change (*r* = −0.09, *p* > 0.05; Figure 4c) or with trough levels of sunitinib plus SU12662 (Figure 5a). However, when comparing trough levels of sunitinib plus SU12662 with a clinical reference of 50 ng/mL, there was a significant change in levels in responsive patients (*p <* 0.001) compared with those in patients who were not responsive to the therapy (*p* = 0.38).

**Table 2:** Change in tumour perfusion, FDG-SUV, clinical benefit, and pharmacokinetic and pharmacodynamic parameters following sunitinib treatment

**Patient**	**Baseline perfusion (mL/min/g)**	**Change in perfusion (%)**	**Baseline FDG-SUV**	**Change in FDG-SUV at week 2 (%)**	**Change in FDG-SUV at week 4 (%)**	**Clinical benefit**^**a**^	**Day 15 trough levels of sunitinib + SU12662**^**b**^**(ng/mL)**	**VEGF plasma levels D15:D1**	**sVEGFR-2 plasma levels D15:D1**
1	0.81	−85	4.6	−39	−48	Yes	76.4	5.29	0.66
2	1.18	−77	6.5	−37	−59	Yes	134.9	4.85	0.44
3	1.52	−59	4.2	−33	−29	No	90.8	8.67	0.34
4	1.28	−34	9.8	−44	−13	No	27.6	2.56	0.75
5	0.74	−69	12.3	−67	−66	Yes	107.1	4.17	0.49
6	1.39	−38	6.0	−32	−18	Yes	88.8	NA	NA
7	1.23	−20	4.1	−29	−41	No	105.0	NA	NA

^a^Clinical benefit was deemed to be present if the treating clinician judged that the patient was benefiting from treatment and continued therapy beyond the 12-week study period. ^b^Target trough level for therapeutic effect was considered > 50 ng/mL. D15:D1, day 15:day 1 ratio; FDG-SUV, ^18^ F-fluorodeoxyglucose standard uptake value; NA, not available; VEGF, vascular endothelial growth factor; sVEGFR-2**,** soluble vascular endothelial growth factor receptor 2.

## Discussion

The results of this study conclusively show that sunitinib treatment of adult patients with a variety of metastatic malignancies is associated with early biological responses, including decreased blood flow in secondary tumour deposits. The reduction in tumour blood flow was associated with a corresponding fall in FDG-SUV, consistent with a decrease in tumour metabolic rate. This is the first study to provide quantitative evidence of the pharmacodynamic effects of sunitinib on tumour blood flow by PET imaging. There was also a possible association between the degree of change in tumour perfusion measured after 2 weeks of therapy and subsequent clinical benefit, duration of therapy, and change in levels of the biomarker VEGF. Blockade of VEGFR signalling by sunitinib may lead to reduced VEGF uptake by VEGFR and, hence, increased circulating VEGF, which was seen in this study.

Not every patient who had a fall in tumour perfusion went on to have a conventional tumour response. Possible explanations for this lack of correlation include a mismatched assessment timing for tumour perfusion (at 2 weeks) and radiological response (at 12 weeks), allowing for tumour progression after an initial early response. Dosing was delayed in one patient, and the dose was reduced in another of the three patients with progressive disease at 12 weeks, which may have influenced the clinical tumour response despite an earlier decrease in tumour perfusion. Mixed responses, with the reference tumour showing a response but other tumour deposits showing progression, and stable or enlarging tumour masses despite central necrosis, could also account for the observed lack of correlation between tumour perfusion and conventional response. In this context, PET imaging may be informative regarding initial pharmacodynamic changes in response to sunitinib treatment, but may not be predictive of the final response.

The methodology used in this study for assessing tumour perfusion changes was based on established techniques and adapted to enable quantitative analysis of tumour perfusion with ^15^O-water-PET without requiring an arterial line. The use of ^15^O-water-PET for the quantitation of blood flow is well established; it is a reliable method to assess the pharmacodynamics of therapeutic agents with a mechanism of action involving angiogenesis inhibition [22]. The short half-life of ^15^O-water does restrict its use to PET centres with on-site cyclotrons. Additional limitations of this study that preclude definitive conclusions include the small sample size and heterogeneous nature of the cases. In addition, there may be a sampling error associated with the selection of target lesions when multiple lesions are present.

Blood flow in solid tumours is often increased relative to the surrounding tissue [23-25]. Moreover, tumour progression beyond a certain size requires active angiogenesis, making antiangiogenic therapy an attractive target for new drug development. Previous studies have indicated a relationship between clinical response, in terms of disease-free survival, and a combination of FDG and tumour perfusion measurements in patients receiving chemotherapy for advanced breast cancer [23]. The role of VEGF-PET as a biomarker of dynamic angiogenic changes has recently been investigated [26]. The effects of sunitinib treatment were assessed using the VEGF-PET tracer ^89^Zr-ranibizumab in mouse xenograft models of human cancer. VEGF-PET demonstrated dynamic changes during sunitinib treatment, with a strong decline in signal in the tumour centre and only minimal reduction in the tumour rim, with a pronounced rebound after sunitinib discontinuation. In addition, VEGF-PET results corresponded with tumour growth and immunohistochemical vascular and tumour markers.

## Conclusions

The results presented here indicate that sunitinib therapy is associated with early biological responses and measurable pharmacodynamic changes in tumour blood flow and metabolism. These data provide important new insights into the antitumour effects of sunitinib in this patient population.

## Competing interests

PLM received funding from Pfizer for a laboratory-based research on the prevalence of *EML4-ALK* mutations in lung cancer patients. RJH received funding support from Pfizer for the Translational Lab and PET Centre via his institution (Peter MacCallum Cancer Centre, Melbourne, Australia). CB, NB, and TJM are Pfizer employees and hold Pfizer stock. GCT acted as a consultant for Pfizer, received honoraria for attending international and Australian advisory board meetings, and received travel sponsorship to attend international meetings, including meetings at which this study was reported; his institution (Peter MacCallum Cancer Centre, Melbourne, Australia) received funding support from Pfizer for the Translational Lab and PET Centre. The other authors have no competing interests to declare.

## Authors' contributions

AMS participated in the design of the molecular imaging components of the study and coordination of molecular imaging data acquisition and analysis. PLM participated in the design and coordination of the study, and analysis. GO'K was involved in the design of the imaging studies and data analysis. TS performed the PET data collection and image analysis. RJH was involved in the conception and design of the molecular imaging components of the clinical trial of which this study represents a subgroup. AP assessed the site of the metabolically active malignancy on the patients' baseline FDG-PET studies and then decided on the tumour site where the ^15^O-water studies would be acquired over and aid in subsequent image analysis. CB was responsible for the clinical development of sunitinib and was therefore involved in the conception, design, and execution of the study, and data evaluation. NB was involved in conceiving of the study and participated in the study design and coordination. TJM reviewed the data and the manuscript. GCT was involved in the conception of the project, protocol writing, patient accrual, and data analysis. All authors were involved in the drafting of the manuscript and approved the final version for submission.
